# Retraction Note: Large-Area Semiconducting Graphene Nanomesh Tailored by Interferometric Lithography

**DOI:** 10.1038/s41598-021-84101-3

**Published:** 2021-02-22

**Authors:** Alireza Kazemi, Xiang He, Seyedhamidreza Alaie, Javad Ghasemi, Noel Mayur Dawson, Francesca Cavallo, Terefe G. Habteyes, Steven R. J. Brueck, Sanjay Krishna

**Affiliations:** 1 Center for High Technology Materials, University of New Mexico, Albuquerque, NM 87106 USA; 2 Department of Mechanical Engineering, University of New Mexico, Albuquerque, NM 87131 USA; 3 Nanoscience and Microsystems Engineering, University of New Mexico, Albuquerque, NM 87131 USA

Retraction of: *Scientific Reports* 10.1038/srep11463, published online 01 July 2015

The Editors have retracted this article following an investigation by the University of New Mexico which established that:

- Figure 2A and 2G contain data points that cannot be substantiated;
- Figure 3A-F and the corresponding histogram data contain images in which contrast has been adjusted or other nonlinear image enhancements made, resulting in images that have smaller feature sizes than were actually present;
- The image shown in Figure S3E does not originate from the same sample as other panels in this figure; Figure S3E appears to also have been manipulated in a manner similar to Figure 3;
- The Raman spectral data in Figures. 4A-C are shifted along the X-axis to produce a systematic shift of the G-band with stated feature size. The spectral data shown in Figure 4C which depict a shift in the G-band peaks irrespective of location on the structures cannot be substantiated;
- Figures 5B and 5C, as well as the red curve in Figure 5D cannot be substantiated;
- In Table I, rows "Min Neck Width (nm)", "ON/OFF Ratio", "Mobility (cm^2^/V.s)", and "Band gap (meV)" in the column "Interferometric Litho" cannot be substantiated;
- Figure 6 and Figure S5 cannot be substantiated.

Sanjay Krishna, Francesca Cavallo and Steven R. J. Brueck agreed with the retraction and its wording. Alireza Kazemi and Terefe G. Habteyes did not respond to the correspondence about the retraction. The Editors were not able to obtain current email addresses for Xiang He, Seyedhamidreza Alaie, Javad Ghasemi, and Noel Mayur Dawson.

